# Topological photonics: robustness and beyond

**DOI:** 10.1038/s41467-024-45194-2

**Published:** 2024-01-31

**Authors:** Alexander B. Khanikaev, Andrea Alù

**Affiliations:** 1 Electrical Engineering Department, The City College of New York (USA), New York, NY 10031 USA; 2 Department of Physics, City College of New York, New York, NY 10031 USA; 3 Physics Program, Graduate Center of the City University of New York, New York, NY 10016 USA; 4 Photonics Initiative, Advanced Science Research Center, City University of New York, New York, NY 10031 USA

**Keywords:** Optical physics, Other photonics

## Abstract

Synthetic optical materials have been recently employed as a powerful platform for the emulation of topological phenomena in wave physics. Topological phases offer exciting opportunities, not only for fundamental physics demonstrations, but also for practical technologies. Yet, their impact has so far been primarily limited to their claimed enhanced robustness. Here, we clarify the role of robustness in topological photonic systems, and we discuss how topological photonics may offer a wider range of important opportunities in science and for practical technologies, discussing emergent and exciting research directions.

Topological photonics leverages the versatility of synthetic optical materials, such as optical waveguide arrays, microring resonator arrays, photonic crystals, metamaterials and metasurfaces, to produce states of light characterized by nontrivial topology of their optical fields. Since the very first prediction of a topologically nontrivial photonic system emulating the quantum Hall effect^1^ and its experimental realization^2^, it has become clear that photonic materials represent an unmatched platform for realizing and testing fundamental concepts of topological physics, and the field of topological photonics has been growing exponentially. The early motivation for this growth was mostly driven by the relative straightforwardness in emulating theoretical models of various topological phases, initially envisioned in condensed matter physics, but often challenging to observe experimentally. Indeed, many exotic topological phases such as the 2-nd Chern number and higher-order topological insulators, including those in synthetic dimensions, were first realized in photonics^3^. Rapidly, topological photonics has established itself as a field of research of its own, driven by the new opportunities offered by topology for photonic applications. The main motivating driver has been the inherent robustness of topological phenomena: since topological features do not change under continuous transformations, topological photonic systems can be expected to possess a degree of robustness to disorder and imperfections that conventional photonic devices may not provide. In addition, photonics offers the opportunity to enrich topological phases with new features, such as nonlinearities, non-Hermiticity, and multiphysics due to strong interactions of light with matter, thus opening new opportunities that go beyond simply emulating condensed matter systems. For instance, topological solitons, non-Hermitian topological phases, and topological polaritons have recently emerged as exciting prospects for topological photonics^3^. Naturally, these exciting discoveries have then translated into opportunities for photonic applications: topological photonic states have been applied for robust guiding and delay lines, controlling the propagation of light via pseudo-spins, and topological lasers^4^, among others.

In this Comment, we discuss the state of topological photonics as a field, questions to be addressed, features and appealing opportunities for emergent science and applications.

## Protected photonic transport

**Fig. 1:**
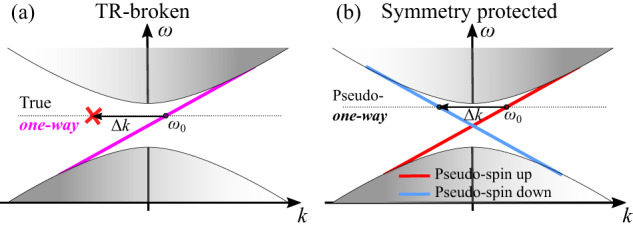
Photonic topological band structures. **a** “True” one-way topological boundary mode (magenta line) with absence of a back-scattering channel for any frequency in the topologically-protected bandgap. For a given frequency ${\omega }_{0}$, any momentum mismatch $\Delta k$ induced by disorder cannot couple to a backward channel. **b** Symmetry protected boundary modes with pseudo-spin-polarized one-way transport, where symmetry reducing defects may lead to spin-flip and back-scattering into the backward mode. $\Delta k$ is the change in momentum due to a defect-induced scattering.

One of the strongest motivations for topological photonics research has been the inherent robustness of topological boundary modes, an idea that was transferred from the field of condensed matter physics. Indeed, robustness to scattering in topological systems with broken time-reversal symmetry (TRS), constitutes a truly unique feature, which is difficult to attain by other means. One-way (Fig. 1a) topological photonic modes can be routed along arbitrary pathways, avoiding backscattering and reflection from defects - an intriguing opportunity for photonic technologies. However, such ultimate resilience can be attained only in systems with broken TRS, e.g., the quantum-Hall-like (QH) topological boundary states^1,2^ and their Floquet counterparts^5^, which ensure truly unidirectional flow of electromagnetic energy with forbidden back-propagation (Fig. 1a). Unfortunately, TRS-breaking in photonics is hindered by practical challenges, because strong magneto-optical effects or temporal modulations are difficult to attain in integrated platforms.

Materials with stronger magneto-optical properties, or materials offering faster and stronger temporal modulation of their dielectric properties may provide routes to enable truly robust topological systems for strong topological protection. In addition, alternative methods of breaking TR symmetry should be explored. As an example, it is known that magneto-optical responses can be strongly enhanced near material resonances, although at a cost of enhanced loss and dispersion. Recent progress in non-Hermitian topological phases^6,7^ shows that one may leverage broken TR symmetry even in lossy regimes, while adding gain could compensate for loss and enhance magneto-optical responses, providing a path towards dissipationless and topologically resilient transport.

Along the same lines, the temporal modulation of material parameters via nonlinear effects may be strongly enhanced near resonances(e.g., near exciton lines), offering a new approach to implement resilient Floquet modes in active nonlinear topological photonic media^8^, while also offering exciting phenomena that may originate from nonlinear responses^9,10^. Systems with polaritonic responses associated with strong light-mater interactions have been successfully used to demonstrate topological polaritonic phases with broken TR symmetry^11^, while enhanced nonlinear phenomena combined with gain hold the promise to enable even more exciting opportunities in this direction – from topological solitons to active all-optical control of topology and of resilient transport.

Nonetheless, while appealing for some applications, strongly protected boundary modes do not necessarily imply a general usefulness from a practical standpoint. While the topological invariant, as a global property of the system, may remain protected after perturbations, this invariance does not imply robustness of all relevant features of the topological modes. For instance, while nontrivial topology ensures backscattering immunity, it does not guarantee preservation of the phase and group velocity, which can lead, for instance, to signal distortion and dispersion. Fortunately, the potential of topological photonics extends far beyond just robustness and backscattering immunity.

### Structured light on a photonic chip spells out symmetry-protected topology

**Fig. 2:**
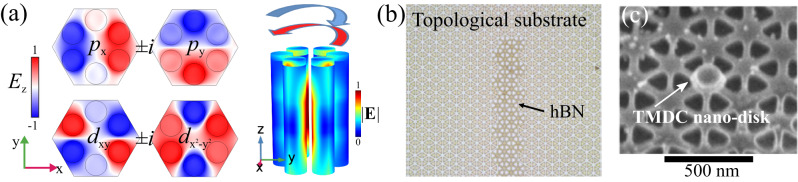
Synthetic pseudo-spin in symmetry-protected topological photonics. **a** Formation of pseudo-spin due to rotational symmetries. Dipolar and quadrupolar modes with rotating field patterns. As in Fig. 1, red and blue rotating arrows indicate PSs “up” and “down”, respectively. **b** A nanopatterned hexagonal boron nitride (hBN) layer laid over a symmetry-protected topological insulator for pseudo-spin-selective light-matter interactions^16^. **c** Precisely integrated nano-disk made of an active polaritonic material (hBN-encapsulated WSe_2_ monolayer) for PS selection-rule engineering.

Due to the existing challenges in breaking TRS, most photonic topological systems thus far have been relying on approaches based on spatial symmetries. Indeed, the symmetries of photonic crystals - sublattice or rotational - enable optical modes endowed with nontrivial spatial modal structure, giving rise to spectral degeneracies that can be treated as components of an effective (pseudo-)spinor (PS). Judicious symmetry reductions then allow to open topological bandgaps hosting PS-polarized boundary modes (Fig. 1b). The valley-Hall^12^ and spin-Hall^13^ topological systems are prime examples of this approach (Fig. 2a). It has always been clear, however, that this symmetry-based approach cannot yield strong resilience to disorder. Indeed, a generic symmetry reduction caused by arbitrary defects or disorder gives rise to pseudo-spin flipping and reflection into the backward mode. Nonetheless, these models have served as an important platform to understand potentials and limitations of symmetry-protected topological phases.

More importantly, these models have led to the realization that lattice symmetry engineering offers much more than just emulation of topological phases, paving new directions to control optical fields at the nanoscale using topological and symmetry concepts^14,15^. Much like structured optical modes propagating in free space, e.g., Gaussian beams carrying orbital momentum, photonic nanostructures with rotational symmetries^16^ support optical modes carrying transverse orbital momentum, a PS degree of freedom (DoF) on chip. While originally used to expand the dimensionality of the available Hilbert space and to engineer the desired topological Hamiltonians, this DoF has now become an important tool to produce structured light on chip and to control light by creating synthetic potentials^16^ (Fig. 2b).

### Light-matter interactions and topological polaritons

An important step in the direction of using synthetic DoFs has been recently made by demonstrating the possibility of selective coupling such pseudo-spins to natural, intrinsic DoFs of matter excitations in topological polaritonic structures^17,18^. At the same time, these early results did not leverage the structure of light to its full extent, and only partial selectivity in light-matter coupling was achieved. The next level of selectivity, which would realize precise selection rules for light-matter interactions, can be achieved by an additional nanopatterning that would align material components with optical fields at the nanoscale (Fig. 2b, c), e.g., with the hotspots of given helicity in the pseudo-spin Hall type of systems^19,20^.

The ability to generate different Hamiltonians spanned by synthetic DoF also brings additional possibilities for mode engineering, giving rise to other topological modes, such as cavity and vortex states, which offer other exciting features for photonic applications, including the generation of vortex beams^21^, resilient multiplexing platforms, and new types of vertically emitting lasers^22^. We believe that further expanding effective Hamiltonians, for instance by coupling to intrinsic DoFs of matter or by leveraging synthetic dimensions, may open even richer physics and more exciting topological phenomena with inherently multi-physics discoveries in which topological photonic phases can be imparted to a wide range of phenomena. In this context, symmetry-protected topological systems are bound to find many exciting applications in photonics and applied technologies, beyond their limitations in terms of robustness.

### Resilience by design

We should also note that the use of terminology borrowed from the condensed matter community, such as robustness of topological states, has been often leading to misconceptions in photonics, with expectations of unreasonable properties for topological states, such as an absolute robustness against defects. Nonetheless, some degree of resilience is certainly present compared to conventional photonic structures, even for the case of symmetry-protected topological systems, provided that the symmetry responsible for the topological phase is not reduced by defects. We note that this is unlikely to be realizable in 2D topological systems, because the in-plane disorder necessarily breaks the topology-defining spatial symmetry of the lattice. However, in quasi-2D systems with preserved dual symmetry, the resilience to defects and disorder was indeed experimentally proven^23^. This makes us believe that new types of symmetries, not limited to real space but also in synthetic dimensions, insensitive to defects in the spatial dimensions of wave propagation, represent another promising approach to realize robust photonic transport rooted in topological order.

## Summary

Despite its relatively long history, topological photonics is still in its infancy in terms of the most exciting scientific and applied prospects. Many unique features enabled by topological phases for photonic applications have only recently raised to the spotlight of the broad optics community. It is apparent that robustness is only one of the many useful aspects enabled by topological properties, and the photonics community should be open to explore broader range of opportunities enabled by topological platforms, both for fundamental physics and for practical applications.
